# Antibody-Based Approaches to Target Pancreatic Tumours

**DOI:** 10.3390/antib11030047

**Published:** 2022-07-12

**Authors:** Marie Sorbara, Pierre Cordelier, Nicolas Bery

**Affiliations:** Centre de Recherches en Cancérologie de Toulouse, Université de Toulouse, Inserm, CNRS, Université Toulouse III-Paul Sabatier, 31100 Toulouse, France; marie.sorbara@inserm.fr (M.S.); pierre.cordelier@inserm.fr (P.C.)

**Keywords:** pancreatic cancer, monoclonal antibody, antibody drug conjugate, imaging, intracellular antibody, KRAS, chemotherapy

## Abstract

Pancreatic cancer is an aggressive cancer with a dismal prognosis. This is due to the difficulty to detect the disease at an early and curable stage. In addition, only limited treatment options are available, and they are confronted by mechanisms of resistance. Monoclonal antibody (mAb) molecules are highly specific biologics that can be directly used as a blocking agent or modified to deliver a drug payload depending on the desired outcome. They are widely used to target extracellular proteins, but they can also be employed to inhibit intracellular proteins, such as oncoproteins. While mAbs are a class of therapeutics that have been successfully employed to treat many cancers, they have shown only limited efficacy in pancreatic cancer as a monotherapy so far. In this review, we will discuss the challenges, opportunities and hopes to use mAbs for pancreatic cancer treatment, diagnostics and imagery.

## 1. Introduction

Pancreatic ductal adenocarcinoma (PDAC) is the most common malignancy of the pancreas (here also referred to as pancreatic cancer). It is a devastating disease with a 5-year overall survival of 11% and is predicted to be the second largest cause of cancer death by 2040 [1]. Several reasons can explain such a poor prognosis: it is usually diagnosed at a late stage, which is often due to non-specific symptoms, a lack of sensitive and specific tumour markers and difficulties in imaging early-stage tumours. In addition, a lack of effective therapies due to the resistance to treatments, such as chemotherapy or radiotherapy, is observed in PDAC. The only potentially curative solution for PDAC patients is surgery, but less than 20% of patients can benefit from a resection [2]. Depending on the stage of the disease, different chemotherapies can be used, including the standard of care for PDAC patients, gemcitabine, a nucleoside analogue approved in 1997 [3]. FOLFIRINOX is a combination of folinic acid (FOL), 5-fluorouracil or 5-FU (F), irinotecan (IRIN) and oxaliplatin (OX) and was approved in 2011 by the Food and Drug Administration (FDA) [4]. Nab-paclitaxel is an albumin-bound nanoformulation of paclitaxel (a taxol derivative) that was approved in 2013 by the FDA in combination with gemcitabine [5]. FOLFIRINOX and nab-paclitaxel (combined with gemcitabine) are mostly used in advanced PDAC. At the molecular level, PDAC is a complex cancer that harbours multiple genetic and epigenetic alterations, making this cancer highly heterogeneous. Based on transcriptomic analyses, PDAC tumours and stroma have been each divided into two predominant molecular subtypes: classical and basal-like for the tumour tissue, normal and activated for the stromal tissue to help stratify patients and find specific therapeutic strategies [6]. However, recent studies showed that PDAC tumours and their microenvironment are more complex and can be classified into several categories [7]. Therefore, understanding the molecular features and the immune landscape of PDAC is essential to develop novel efficient (targeted) therapies. This is exemplified with the FDA approval of PARP inhibitor olaparib for patients with germline *BRCA1* or *BRCA2* mutations in platinum-sensitive metastatic PDAC [8], further demonstrating the importance of deciphering the tumours at the molecular level. However, multiple hurdles need to be overcome in PDAC to improve patients’ outcomes, such as fighting therapeutic resistance, discovering early detection methods and effective therapeutics.

**Figure 1:**
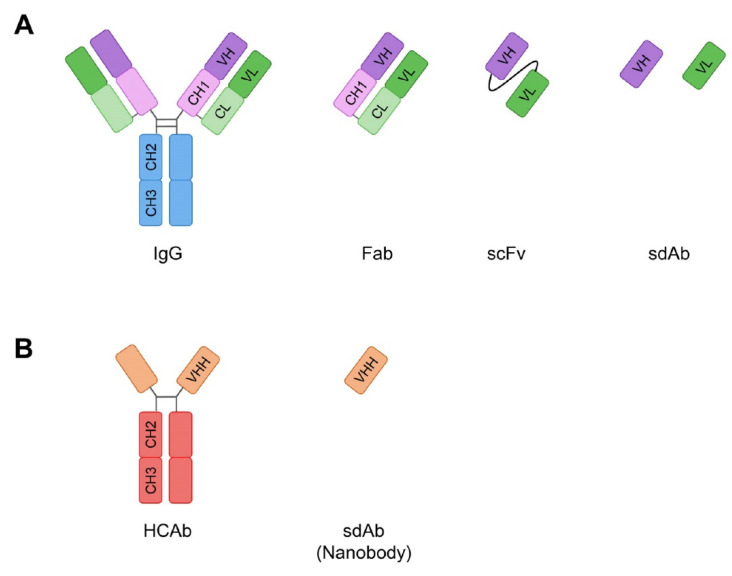
Different antibody formats. (**A**) Full-length immunoglobulin G (IgG) and its derivatives Fab (fragment antigen-binding), scFv (single chain fragment variable) and sdAb (single domain antibody). VH (variable heavy) and VL (variable light) are the domains that enable the binding to the antigen. (**B**) Heavy chain only antibody (HCAb) found in Camelidae and its derivative VHH (variable heavy domain of heavy chain only antibody) that is a sdAb and is also called nanobody.

Monoclonal antibodies (mAbs) are an attractive avenue to answer these challenges in PDAC. They exist in different formats, such as full-length immunoglobuline (Ig) or as antibody fragments (e.g., fragment antigen binding (Fab), single chain fragment variable (scFv), single domain antibodies) (Figure 1). Each of them has advantages/drawbacks depending on their final application. For instance, larger antibody formats (i.e., full size or Fab) will usually have a longer half-life in the bloodstream compared to smaller fragments (scFv or single domains), but the latter could penetrate into tumours more easily due to their small size. Antibodies are versatile molecules that can be selected in vitro by phage display and used as direct blocking therapeutics for intracellular or extracellular proteins [9,10]. When conjugated, they can deliver a drug payload, such as antibody drug conjugate (ADC) [11] or radiolabelled antibodies, for targeted radionuclide therapy [12]. Finally, if functionalised with a fluorescent or radio-analogue moiety, mAbs can be used as an imaging agent.

Here, we review the potential of antibodies to target cell surface or intracellular proteins in PDAC but also how they can be modified to kill cancer cells or specifically detect tumours.

## 2. Targeting the Surfaceome of Pancreatic Tumours

**Figure 2:**
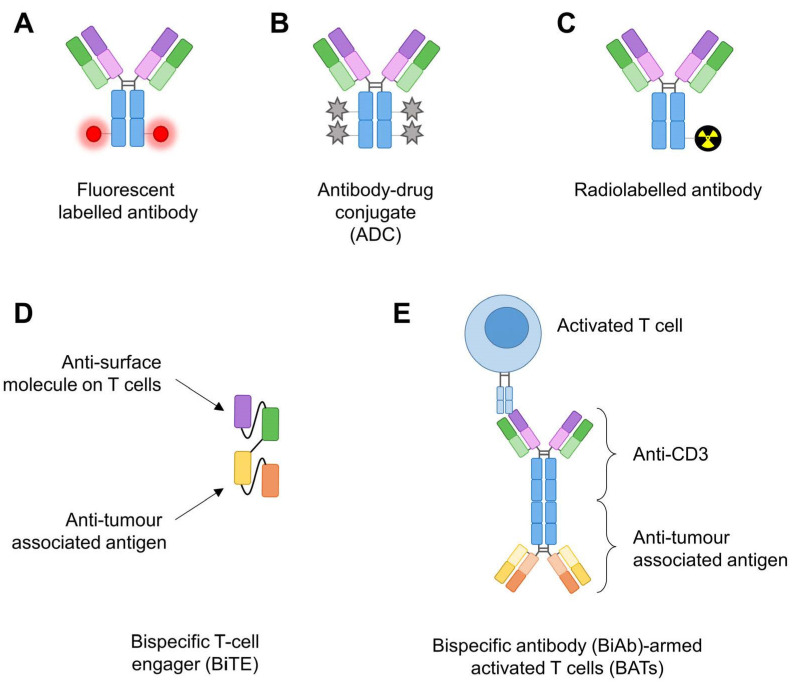
Antibodies’ functionalisation for cancer therapy. (**A**) Fluorescent labelled antibody: a fluorescent dye (usually far-red) is linked to an antibody. (**B**) Antibody-drug conjugate (ADC) is an antibody modified with cytotoxic drugs. (**C**) Radiolabelled antibody is linked to a radioisotope moiety for targeted radionuclid therapy and/or imaging. (**D**) Bispecific T cell engager (BiTE) is usually two scFvs linked together: one binding a tumour-associated antigen (TAA) and the other one a surface molecule on T cells. (**E**) Bispecific antibody-armed activated T cells (BATs) correspond to two full-length IgGs crosslinked and incubated with autologous activated T cells (i.e., from patients), and the BATs are reinfused back into the patients.

Around 25–30% of human genes encode for cell surface proteins, also called surfaceome [13]. Some membrane-bound proteins have been successfully targeted by antibodies to directly block downstream signalling pathways or, when functionalised with molecules, to image tumours for diagnostic or surgery or to specifically kill cancer cells (e.g., ADC or targeted radionuclid therapy) (Figure 2).

### 2.1. EGFR

EGFR belongs to the family of transmembrane tyrosine kinase receptors that includes HER receptors. It is mutated and also overexpressed in numerous cancers, including pancreatic cancer, making this receptor a key therapeutic target. EGFR-mediated signalling plays a role in proliferation, metastasis and apoptosis evasion [14]. Therapeutic targeting of EGFR by erlotinib, a tyrosine kinase inhibitor, combined with gemcitabine has shown modest but reproducible responses in patients with unresectable metastatic pancreatic cancer, leading to its approval by the FDA [15]. Several preclinical studies using pancreatic cancer xenograft in nude mice have supported the strategy to disrupt EGFR-mediated signalling with cetuximab [16], a monoclonal chimeric IgG1 that targets the receptor protein expressed on the cell surface [17]. In addition, combination of gemcitabine and cetuximab in an orthotopic PDAC mouse model showed an additive anti-tumour effect [18]. These studies, added to the success of cetuximab in colorectal cancer, prompted the investigation of anti-EGFR therapies in PDAC patients. However, the phase III study results were disappointing and showed that gemcitabine plus cetuximab treatment did not improve the patients’ outcomes compared with patients treated with gemcitabine alone [19]. Furthermore, patients in the cetuximab/gemcitabine arm experienced more frequently grade 4–5 toxicities compared to the gemcitabine arm [19].

While targeting EGFR with cetuximab and chemotherapy was not successful, the anti-EGFR therapies focus is now on repurposing their use in pancreatic cancer. Consequently, anti-EGFR antibodies have been functionalised with molecules to either monitor, target or kill, more specifically, pancreatic tumours. Near-infrared (NIR) fluorescence is a promising technology to help visualise the tumour during surgery. Anti-EGFR antibody panitumumab was linked to a fluorescent fluorophore (IRDye800CW, Figure 2A), and a clinical trial was performed in patients with pancreatic cancer undergoing surgery (NCT03384238). This study showed this fluorescent tracer is safe and feasible to use during PDAC surgery [20]. A humanised anti-EGFR antibody-based ADC (Figure 2B) was developed by conjugating monomethyl auristatin E (MMAE), a microtubule destabiliser, to the antibody. This ADC is specific to EGFR expressing cells, is only cytotoxic in these cells in vitro and showed anti-tumour activity in vivo [21], but it is still not tested in clinical trials. Cetuximab was used as a targeting agent for camptothecin (CPT, a chemotherapy) encapsulated into polymeric nanoparticules. Cetuximab nanoconjugation enhanced the CPT delivery in vitro and improved the growth-inhibitory effects in vivo [22]. Cetuximab was also conjugated to murine IgG2a anti-CD3 mAb (OKT3) to make a bispecific antibody. Activated T cells were armed with this bispecific Ab (EGFR BATs, Figure 2E) to enhance receptor-directed cytotoxicity [23]. EGFR BATs are ex-vivo-expanded autologous activated T cells armed with a bispecific Ab that are reinfused back into the patient. These engineered T cells showed an anti-tumour effect in preclinical settings [23], and the first results of two clinical trials involving seven patients (NCT01420874, completed phase Ib; NCT02620865, completed phase II) are encouraging. Actually, infusions of BATs in patients are safe (only toxicities grade 1–3 side effects and no dose-limiting toxicities), induced anti-pancreatic cancer cytotoxicity with immune and cytokine responses and a median overall survival (OS) of 31 months for the seven patients [24]. These promising results will need to be confirmed with a larger cohort of patients.

Even though EGFR antibodies showed no benefit to treat PDAC patients, a study by Blasco and collaborators showed that genetic removal of EGFR and CRAF kinase in a mouse model induced the regression of PDAC tumours in vivo, suggesting that a combination of EGFR and CRAF inhibitors could be of interest for PDAC treatment [25], but this has still to be assessed.

### 2.2. Mesothelin

Mesothelin (MSLN) is a 40 kDa glycosyl phosphatidylinositol anchored cell surface protein expressed on mesothelial cells. The physiological role of MSLN remains unknown, but it seems non-essential because mice harbouring a null mutation in the mesothelin gene exhibit normal development and reproductive capabilities [26]. Interestingly, normal pancreatic tissue does not express RNA coding for the precursor of mesothelin nor mesothelin protein, but it is overexpressed in several cancers, including PDAC [27]. In addition, overexpression of MSLN in PDAC is found in almost all tumours, which is interesting knowing the particularly high heterogeneity of these tumours. All those characteristics point towards MSLN as a target of interest in PDAC.

Hence, a recombinant anti-mesothelin immunotoxin, named SS1P, was developed by fusing a mouse anti-MSLN disulfide-stabilized Fv antibody fragment (dsFv, named SS1) to a truncated fragment of Pseudomonas exotoxin A (PE) [28]. The preclinical data using this antibody-based therapy were promising as combination of treatment of SS1P with radiation, taxol or gemcitabine resulted in enhanced antitumor activity against mesothelin-expressing tumour xenografts [29,30,31]. Then, a phase I clinical trial that included two PDAC patients was conducted by treating patients with SS1P, but the results showed modest clinical activity. Actually, 88% of the patients developed neutralising antibodies against SS1P due to the high immunogenicity of PE, thus limiting the treatment to only one cycle of therapy [32]. To overcome this immunogenicity in patients, Mossoba et al. showed, in a proof-of-concept preclinical study, that an immune depletion regimen could abrogate anti-immunotoxin reactivity [33]. Therefore, a phase II study that consisted of SS1P treatment combined with pentostatin and cyclophosphamide, two drugs that help suppress the immune system, was initiated. Unfortunately, around 20% of the patients still developed neutralising antibodies and none of the PDAC patients completed the trial because of adverse events or progressive disease (NCT01362790). After the failure of SS1P, a second generation of immunotoxin was designed with a low-immunogenic modified PE fragment fused to a humanised anti-mesothelin Fab fragment and named RG7787 and later on LMB-100 [34]. Preclinical studies of RG7787 in combination with paclitaxel in pancreatic cancer showed durable anti-tumour responses [34]. However, it is worth noting that this immunotoxin could only reach 45% of the tumour in vivo [34], highlighting the barrier role of the PDAC dense stroma. A follow-up study showed the synergic effect in a PDAC mouse model treated with LMB-100 and nab-paclitaxel with complete regression of tumours treated by this combination [35]. These data led to a clinical trial of LMB-100 combined with nab-paclitaxel on patients with advanced PDAC (NCT02810418). Although clinical activity was observed, the combination was not well tolerated by patients [36]. Anetumab ravtansine, an ADC of anti-MSLN antibody linked to maytansinoid DM4 drug, was tested in a phase I study in metastatic solid tumours, including pancreatic cancer (NCT01439152) [37]. Anetumab ravtansine showed manageable safety with encouraging anti-tumour activity [37]. A phase II study followed and is completed, but the data are pending (NCT03023722).

Amatuximab or MORAb-009 is a chimeric anti-mesothelin Ab that was developed by grafting the mouse VH and VL fragments of SS1 with human IgG1 and kappa constant regions. This Ab elicited antibody-dependent cellular cytotoxicity (ADCC) on pancreatic cancer cells in vitro and blocked the interaction between MSLN and its ligand CA125/MUC16 [38]. Interestingly, this interaction is thought to facilitate metastasis of cancer cells [39]. Preclinical studies showed promising results by enhancing the anti-tumour effects of gemcitabine and taxol in vivo [38,40]. The results of phase I studies revealed that MORAb-009 is well tolerated [41] and led to phase II studies with MORAb-009 in combination with gemcitabine (NCT00570713). However, this trial was not completed because of a lack of efficacy in more than 50% of the patients (no improvement in OS or progression-free survival (PFS) compared to the control group gemcitabine only). Moreover, amatuximab was tested alone in another clinical trial (NCT01413451) on patients with cancers expressing high levels of mesothelin (including PDAC patients). Even though no results were published, a differential biodistribution of MORAb-009 uptake level was shown that is higher in mesothelioma patients compared to PDAC patients using single photon emission computed tomography-computed tomography (SPECT-CT) imaging [42]. This echoes to other studies discussed above and highlights again the issue for biologics to go through the dense tumoral microenvironment (TME) of PDAC tumours. Overall, these studies show how difficult it is to translate encouraging preclinical data into the clinic. Nevertheless, functionalising anti-MSLN mAb as ADC [37] or using anti-MSLN scFv in a chimeric antigen receptor T cell (CAR T cell, reviewed in Ref. [43]) therapy [44,45] might be a solution to achieve efficacy in patients.

### 2.3. Mucins

Mucins are a family of multifunctional glycoproteins expressed on the surface of epithelial cells in the gastrointestinal tract that are playing pivotal roles in gut lubrication and protection. Almost all proteins among this family are globally overexpressed in pancreatic cancer [46]. Actually, they form a protective coat around cancer cells and have important roles in the carcinogenesis of PDAC [47]. They are involved in many malignant processes, including evasion, invasion and metastasis, by affecting oncogenic signalling, cell survival, proliferation and resistance to chemotherapeutics [48]. Moreover, mucins in PDAC present a specific pattern of expression during the different steps of tumour progression toward carcinoma [46]. This family of proteins, and particularly MUC1/4/5, have drawn attention and investigation, such as new biomarkers and therapeutic targets in PDAC, notably with antibody-based therapy [49].

Gatipotuzumab, also known as PankoMab-GEX, is a humanized IgG1 anti-MUC1 mAb that binds with high affinity to a novel carbohydrate-induced conformational epitope on MUC1 (named tumour-related MUC1 epitope, TA-MUC1) [50]. TA-MUC1 is highly expressed in a broad variety of carcinomas and virtually not expressed on normal cells. This mAb is, therefore, displaying numerous advantages compared to other anti-MUC1 antibodies in clinical development with higher tumour specificity, higher affinity and rapid internalisation [50]. Hence, Gatipotuzumab was tested in phase I clinical studies on patients with advanced carcinomas, including PDAC (NCT01222624). The data revealed that the mAb is safe, well tolerated, and showed promising anti-tumour activity in advanced disease. Of note, adverse events were mainly mild-to-moderate infusion-related reactions in about 50% of the patients. However, there was no efficacy on pancreatic cancer patients [51], and this mAb is still not approved for clinical use. Because Gatipotuzumab internalises rapidly in cancer cells, this property prompted the companies Glycotope and Daiichi Sankyo to develop an ADC version of this mAb that is currently under preclinical assessment [52].

Another anti-MUC1 mAb was developed, PAM4, that shows high specificity for MUC1 expressed by PDAC compared to other cancers, normal pancreas or pancreatitis [53]. Taking advantage of its high specificity, the humanised version of this mAb was radiolabelled (Figure 2C) and used for either nuclear imaging when PAM4 was labelled with an indium 111 (^111^In) radioisotope or for targeted radionuclid therapy when PAM4 was functionalised with yttrium-90 (^90^Y) radioisotope [54]. ^90^Y-PAM4 showed anti-tumour response with reasonable adverse effects in vivo [55]. Therefore, these results prompted the use of humanised PAM4 antibody labelled with the aforementioned radioisotopes in an early phase I clinical study in patients with PDAC (NCT00364364). Unfortunately, this clinical trial was terminated without any data due to loss of funding.

PAM4 mAb is selective of MUC1 expressed by PDAC; hence, its ability to detect the early stage of PDAC was tested by immunohistochemistry. While another anti-MUC1 antibody, MA5, stained several normal tissues (e.g., pancreas, colon, stomach, lung) and many tumour tissues, PAM4 did not, confirming its PDAC specificity. In addition, PAM4 labelled 94% of the earliest PanIN lesions (PanIN-1A, -1B) by immunohistochemistry [56], showing promising results to employ this mAb for the detection of early-stage disease. Finally, an anti-TA-MUC1 mAb (5E5) was developed [57] and employed successfully in a CAR T cell therapy setting in a preclinical study [58].

MUC4 is not expressed in healthy pancreas, but it is overexpressed in 70–80% of pancreatic cancer [59]. Interestingly, MUC4 expression is linked to resistance to treatment in PDAC, including gemcitabine, the standard of care for PDAC patients [60]. Hence, targeting MUC4 could be useful for tumour detection and therapy, but the development of therapeutic mAbs is required. MUC5AC is also overexpressed in PDAC, with minimal expression in healthy pancreatic tissue, which makes it a targetable marker in this cancer [61]. Therefore, several groups used or developed anti-MUC5AC mAbs for therapy or tumour detection. When linked to ^111^In [62] or ^89^Zirconium (Zr) [63] radioisotopes or to a NIR dye (IRDye800CW) [64], anti-MUC5AC mAbs could preferentially target pancreatic cancer tissue compared to normal pancreatic tissue. In the future, fluorescent mAbs could help guide the resection of PDAC. A humanised anti-MUC5AC antibody was labelled with ^225^Actinium (Ac) for targeted radionuclide therapy and demonstrated efficacy to suppress tumour growth in mice [63]. However, none of these MUC5AC antibodies are currently in clinical trials.

### 2.4. CEA

Carcinoembryonic antigen (CEA), or CEACAM-5, is a cell adhesion molecule anchored to the cell membrane involved in extracellular matrix adhesion, motility and inhibition of apoptosis [65]. It plays a crucial role in a number of biological processes, including homeostasis, embryogenesis and development of neural tissue, inflammation, immune cell transmigration and immune response. However, CEA is not expressed in healthy pancreas and moderately expressed in pancreatitis, strengthening its potential as a PDAC-specific target [66]. After carbohydrate antigen CA19-9, CEA is the second most used biomarker for diagnosis and monitoring of PDAC. As a matter of fact, CEA serum levels are increased in 40–70% of all PDAC patients, while CEA is overexpressed on the cell membrane in 70–85% of PDAC cases [67]. Recently, a retrospective analysis showed that CEA before neoadjuvant chemoradiotherapy is a crucial prognostic indicator for localised PDAC [68]. Therefore, mAbs were developed to target CEA for image-guided surgery to detect and/or inhibit PDAC tumours but also as anti-CEA CAR T cell therapy (NCT03818165). However, this clinical trial was terminated as a consequence of a limited number of recruited patients. A chimeric fluorescent ADC anti-CEA mouse antibody was tested for its ability to detect and inhibit tumour growth in vivo [69]. This mAb was linked to paclitaxel and an infrared fluorophore (DyeLight680) and showed the feasibility to (i) localise the tumour with long-lasting effect and to (ii) impede tumour growth in vivo [69]. Nevertheless, it is important to note that paclitaxel is not a drug approved for PDAC therapy and that the mouse origin of the antibody could limit its direct use in patients. Therefore, additional optimisation steps of this fluorescent ADC are required before a potential clinical application. However, an ADC anti-CEA humanised mAb (tusamitamab ravtansine) is currently under clinical trial for patients with metastatic PDAC and breast cancer (phase II, NCT04659603). The same mAb was designed as a fluorescent molecule when linked to IRDye800CW and showed promising results to probe human pancreatic cancer in vivo with a favourable tumour-to-background ratio [70].

So far, the only fluorescent mAb that is in clinical trial is SGM-101, an anti-CEA chimeric mAb that is engineered with an original NIR dye (BM104). This fluorescent tracer specifically labelled the tumours from an orthotopic pancreatic cancer model in vivo using BxPC-3 cells with a tumour-to-background ratio of 3.5 [71]. Based on these encouraging results, a phase I clinical trial was performed (NCT02973672) and showed the use of SGM-101 is safe (no adverse events were observed in patients except one with diarrhoea) and feasible for the detection of both primary PDAC and metastasis [72]. While SGM-101 is now tested in several phase II clinical studies for colorectal cancer metastasis (NCT04737213, NCT03659448, NCT04755920), it is still not the case for pancreatic cancer. This is probably because additional prospective research is needed to test whether this technique will ultimately improve OS of PDAC patients. Moreover, image-guided surgery of PDAC tumours could also require the use of other agents targeting the tumour stroma, abundant in pancreatic cancer, to increase sensitivity [72]. Full-length mAbs are useful for image-guided surgery because of their long half-life fluorescence signal in the tumour. However, it takes 2 to 3 days to obtain the tumour fluorescence because the accessibility of the tumour by the mAb is limited by the antibody’s size. Hence, developing smaller antibody fragments, such as sdAbs (e.g., nanobodies), could circumvent this drawback. Recently, an anti-CEA nanobody conjugated to an IR800CW dye was developed to target and label patient-derived pancreatic cancer xenografts in mice. It efficiently reached the tumour within an hour with a good tumour-to-background ratio (of 2.0 by 3 h) and allowed a durable fluorescence signal over hours. These characteristics make this fluorescent nanobody a promising and practical molecule for precise fluorescence-guided surgery of pancreatic cancer [73]. Interestingly, if the anti-CEA nanobody targets another epitope than the mAb, a combination of both fluorescent mAb and nanobody could enable a more rapid fluorescence of the tumour within hours to accelerate the patients’ care with a durable fluorescence signal useful for long surgery, such as pancreatic cancer resection.

### 2.5. Exploring the Surfaceome for the Discovery of Novel Targets and Biomarkers

**Figure 3:**
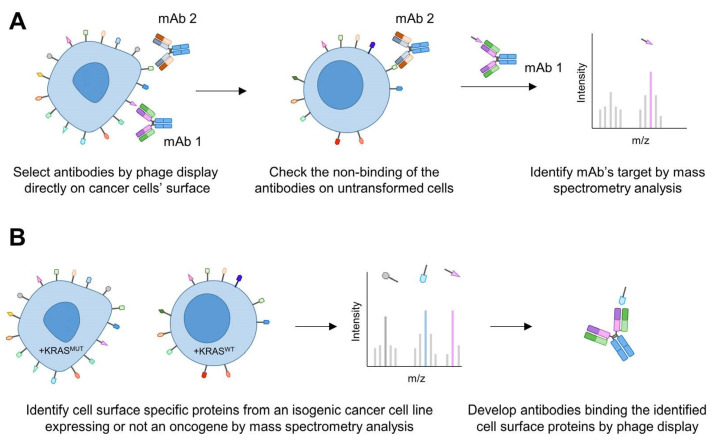
Discovery of novel cell surface proteins with antibodies. (**A**) Selection of mAbs directly binding to cancer cells by phage display and that do not bind to untransformed cells. Next, their target is identified by mass spectrometry analysis. (**B**) Determination of the surfaceome of cancer cells expressing an oncogene (e.g., mutant KRAS, KRAS^MUT^) compared to the same cell line not expressing the oncogene (e.g., wild-type KRAS, KRAS^WT^) to discover oncogene-dependent cell surface proteins by mass spectrometry analysis. mAbs are then selected by phage display against these newly discovered proteins.

As discussed above, only a few membrane proteins have been targeted in pancreatic cancer; therefore, the discovery of novel (pancreatic) cancer-specific targets is necessary. To this end, two different methods have been implemented for the development of membrane-protein-specific antibodies (Figure 3).

The first approach consisted of isolating mAbs binding to the surface of cancer cells by phage display (Figure 3A). The mAbs were screened directly on intact cells from seven different carcinomas, including pancreatic cancer cell lines [74]. Then, the antibody clones of interest were selected using fresh tumour tissues by immunohistochemistry. If the clones were binding to malignant cells and not or weakly to normal cells (by checking their staining on the respective type of cells), they would be further characterised by mass spectrometry to identify their target. Using such an approach, a mAb targeting CD147, a transmembrane protein overexpressed in tumours including pancreatic cancer, was shown to induce ADCC and to inhibit the growth of PANC-1 pancreatic cancer cells [74,75]. This antibody was also radiolabelled to monitor pancreatic cancer cells in vivo [76].

In a second approach, the research of novel cell membrane targets was performed first by determining the specific surfaceome of cancer cells. This has been successfully applied to Ewing sarcoma [77] and T cell acute lymphoblastic leukemia (T-ALL) [78] to find cancer cell surface targets and enabled the development of an ADC to selectively kill cancer cells [77].

While the surfaceomes from these studies were determined by RNAseq analysis of cancerous versus non-cancerous cells, another method was developed and used isogenic cell lines [79]. These cell lines allowed the discovery of the cell membrane proteins specifically expressed upon oncogenic KRAS^G12V^ expression compared to KRAS^WT^ cells by mass spectrometry analysis. After finding the KRAS-regulated surfaceome proteins, recombinant monoclonal antibodies were generated by phage display against seven different membrane-bound proteins, including CUB domain containing protein 1 (CDCP1) (Figure 3B). CDCP1 drives loss of adhesion through integrin signalling [80]. It is overexpressed in various cancers and has been previously involved as a driver of cancer cell growth, metastasis and tumour progression [81]. It is specifically expressed on pancreatic cancer cell lines and not on non-tumorigenic pancreatic ductal cells (HPNE), making this protein an attractive target. Because antibodies are versatile tools, anti-CDCP1 antibody was engineered and used in various set-ups. Notably, it enabled the delivery of an ADC to selectively kill PDAC cells. When engineered in a bispecific T cell engager (BiTE) modality (fused to an anti-CD3 scFv, Figure 2D), the antibody could recruit and activate T cells to PDAC cells while sparing normal cells. Finally, when labelled with a positron-emitting radioisotope (^89^Zr), the antibody showed efficacy to image the tumour in vivo [79]. This example highlights the power of this approach to discover novel antibodies against membrane proteins that have an expression controlled by an oncoprotein.

## 3. Targeting Immune Checkpoints

Immune checkpoints are receptors expressed by immune cells that enable dynamic regulation of immune homeostasis and are particularly relevant to T cell functionality. The most studied receptors are programmed cell death protein 1 (PD-1)/programmed cell death ligand 1 (PD-L1) and cytotoxic T-lymphocyte-associated antigen 4 (CTLA-4)/CD80 (also known as B7). When the receptors are in interaction, this leads to T cell exhaustion (i.e., an “inactive” state). This phenomenon is found physiologically to limit autoimmune inflammation or maintain foetal tolerance during pregnancy but is also exploited by cancer cells to maintain immune tolerance.

Therefore, immune checkpoint inhibitors (CPIs) have been developed to inhibit these receptors’ interactions and consequently reactivate the immune system (e.g., T cells) to modulate the immune response against cancer cells [82]. In 2011, ipilimumab, the first antibody blocking an immune checkpoint (CTLA4), was authorized by the FDA. This was rapidly followed by the development of monoclonal antibodies targeting PD-1 (pembrolizumab and nivolumab) and PD-L1 (atezolizumab and durvalumab), which impede PD-1/PD-L1 interaction [83]. T-cell-targeted immunomodulators are now used as single agents or in combination with chemotherapies as first or second lines of treatment for various cancers. This strategy was successful across numerous solid tumours, such as melanoma, non-small cell lung cancer, renal cancer, hepatocellular carcinoma and mismatch repair-deficient metastatic colorectal cancer, producing sustained anti-tumour responses [84]. We will now review in this section the advances and challenges in pancreatic cancer immunotherapy.

Compared to normal pancreatic samples, PD-L1 expression is upregulated in 19% of tumour samples, and it was suggested that CPIs might reactivate exhausted T cells to increase the anti-tumour immune response in PD-L1-upregulated tumours [85]. In addition, CTLA-4 is widely expressed within the TME. In tumour lesions, it is found expressed on infiltrating Tregs, conventional exhausted T cells, or on tumour cells themselves, contributing to an immunosuppressive environment [86,87,88]. Furthermore, blockage of CTLA-4/CD80 interaction is sufficient to induce CD4+ T cell infiltration into pancreatic tumours, demonstrating that the CTLA-4/CD80 axis regulates T cell infiltration in pancreatic cancer [88].

However, the early-phase clinical studies employing either PD-1 [89], PD-L1 mAbs [90] or CTLA-4 mAbs [91] as monotherapy did not show any clinical benefit. These negative clinical studies highlighted the resistance of PDAC to CPIs. This resistance can be explained by the complex immunosuppressive landscape of the PDAC microenvironment. It notably impedes tumour infiltration by effector T cells, making immune quiescent tumours (also called immunologically “cold” tumour) [92], while PDAC cells harbour neoantigens of high quality [93] that are immunoedited with time in long-term survivors [94]. Hence, the focus was next to fight this immunosuppressive TME by using various combination therapies, including dual CPIs therapy, CPIs with chemotherapies, radiotherapies or vaccines [95].

Chemotherapies were initially thought to prevent tumour growth by inhibiting cellular proliferation or inducing cell death. However, recent studies indicate that chemotherapeutic drugs can boost the immunogenicity of tumour cells or cause immunogenic cell death (ICD) in various tumour models [96]. On one hand, gemcitabine can affect the TME through the inhibition of the expansion of immunosuppressive cells, such as myeloid-derived suppressor cells (MDSCs) [97]. On the other hand, it can also induce the expansion of anti-inflammatory M2 macrophages by a T helper 2 cytokine environment [98], which have been shown to increase the resistance to gemcitabine by upregulating cytidine deaminase level [99]. Hence, ipililumab was tested in a clinical trial (NCT01473940) in combination with gemcitabine and nab-paclitaxel, where its efficacy was comparable to chemotherapy alone [100]. However, combining pembrolizumab, gemcitabine and nab-paclitaxel slightly improved the OS of PDAC patients (NCT02331251) [101]. The main difference between these clinical trials is that the pembrolizumab trial was conducted on chemotherapy naïve patients, while the ipililumab-based trial was not.

Focal adhesion kinase (FAK) is an important regulator of the fibrotic and immunosuppressive TME in PDAC [102]. Its inhibition increased immune surveillance by defeating the stromal fibrosis and immunosuppressive PDAC TME and induced sensitisation to immunotherapy [102]. Therefore, pembrolizumab and a FAK inhibitor, defactinib, are used in clinical trials together (NCT02758587) or combined with gemcitabine (NCT02546531). So far, the results are not available. The same type of clinical trials are ongoing with different chemotherapies and/or antibodies (nivolumab/nab-paclitaxel (NCT02309177), nivolumab/FOLFIRINOX (NCT03970252)), and the outcomes are not published yet.

Interestingly, knowing the benefit of olaparib treatment in platinum-sensitive metastatic PDAC patients with germline *BRCA1/2* mutations, a new clinical trial was launched in 2021 to study the combination of pembrolizumab with olaparib in patients with metastatic PDAC with a high tumour mutation burden (NCT05093231).

Radiotherapy (RT) is known to induce ICD [103] and an abscopal effect. This occurs when RT not only shrinks the targeted tumour but also induces tumour regression at non-irradiated, distant sites [104]. Additionally, the combination of local RT and immune-modulation could increase local tumour control and cause distant anti-tumour effects through increased tumour-antigen release and antigen-presenting cell (APC) cross-presentation, improved dendritic-cell (DC) function and enhanced T cell priming [105,106,107]. Actually, preclinical studies showed that RT induces an abscopal tumour-specific immune response in both the irradiated and non-irradiated tumours that is potentiated by PD-1 blockage [108]. A synergic effect of RT and anti-PD-L1 was demonstrated on PDAC mice models [109]. Moreover, in the same study, the authors indicated that RT induces immunosensitisation of tumour cells and that anti-PD-L1 increases recruitment of CD8+ T cells and decreases the establishment of suppressive microenvironment factors [109]. Accordingly, clinical trials evaluate the efficacy of these different combinations of CPI and RT in pancreatic cancer. For example, a phase II trial combining RT, ipilimumab and nivolumab in patients with metastatic microsatellite-stable colorectal or PDAC (25 patients) was performed (NCT03104439). It demonstrated the safety but also the modest efficacy of RT to enhance the effects of dual checkpoint inhibition in MSS metastatic CRC and PDAC [110]. While combining chemotherapy or RT with CPIs only showed limited improvement for the patients so far, studying the potential efficacy of dual checkpoint inhibition (ipilimumab, nivolumab) in combination with gemcitabine and nab-paclitaxel followed by immune-chemoradiation in locally advanced pancreatic cancer (LAPTOP, NCT04247165) might be the solution to obtain a clinical benefit.

Lastly, combination of CPI with vaccines is another therapeutic strategy under investigation in PDAC patients. Indeed, vaccines may have the potential to convert “non-immunogenic” PDAC into an immunogenic tumour through enhanced antigen presentation and priming of antigen-specific T cells. Several vaccines are tested, including GVAX, which is a whole tumour cell vaccine genetically engineered to express granulocyte-macrophage colony-stimulating factor (GM-CSF) [111]. It consists of two irradiated human allogeneic pancreatic tumour cell lines modified to secrete GM-CSF, a cytokine that induces the maturation of dendritic cells. Initially tested as a single agent in cancers, it is now under investigation in combination therapies, including with CPIs. Combination of GVAX vaccine and PD-1 blocking antibody facilitates effector T cell infiltration into pancreatic tumours and, consequently, improved murine survival compared to PD-1 antibody monotherapy or GVAX therapy alone [112]. Accordingly, different clinical trials studied the most efficacious combinations. Unfortunately, the results of the phase II STELLAR trial, where the cancer vaccine GVAX, cyclophosphamide (CY) and CRS-207 (live, attenuated *Listeria monocytogenes* expressing mesothelin), evaluated with or without nivolumab, were disappointing (NCT02243371). Neither improvement to OS (5.88 vs. 6.11 months) nor significant differences for PFS or time to progression were shown in this clinical study.

Anti-CTLA-4 mAb, ipilimumab, was administered in locally advanced or metastatic pancreatic cancer in combination with GVAX vaccine (NCT00836407). Clinical activity was observed with improved OS (5.7 versus 3.6 months) and 1-year survival (27% versus 7%) in patients that received ipilimumab and GVAX versus ipilimumab alone, indicating the potential efficacy of this combination [113]. However, giving combination GVAX and ipilimumab immediately after front-line chemotherapy (here FOLFIRINOX) in the maintenance setting did not improve OS, but biological effects on immune cells were observed (NCT01896869) [114]. Further study of novel combinations in the maintenance treatment of metastatic PDA is feasible.

Recently, a phase I trial used a sequential treatment: tumour resection, followed by atezolizumab (PD-L1 blockade), followed by a systemic mRNA-based personalised neoantigen-specific immunotherapy vaccine (called autogene cevumeran) and FOLFIRINOX chemotherapy (NCT04161755). The preliminary results recently released are promising: out of 19 patients, 16 received the vaccine and 50% had neoantigen-specific immunity that correlates with improved a PDAC outcome compared with non-responders [115]. These data showed autogene cevumeran is safe, feasibly manufactured in a clinically relevant timeframe and immunogenic in PDAC [115]. The individualised neoantigen tumour vaccines are probably one solution for PDAC patients knowing the high heterogeneity between the patients’ tumours.

The future of immunotherapy will probably rely on the implementation of a personalised vaccine but also on the development of novel agents targeting additional immune checkpoints, co-stimulatory receptors and/or co-inhibitory receptors that control T cell function to improve the efficacy of the current CPIs [116]. In addition, analysis of the tumour-infiltrating lymphocytes (TILs) in PDAC biopsies could help determine the tumour immune status in order to select patients suitable for immunotherapy [117].

## 4. Targeting Intracellular Proteins

Although PDACs are highly heterogeneous tumours at both the inter- and intra-tumoral genomic level, recurrent genetic and molecular alterations are common traits of this cancer. Those include activating mutations on *KRAS* (>90% of tumours) [118] and inactivating mutations of *TP53*, *CDKN2A* and *SMAD4* (50–80%) [2]. Therefore, such proteins could be attractive therapeutic proteins to inhibit or stabilise. However, these are intracellular proteins that remain difficult to target with small molecules, with the exception of the KRAS^G12C^ mutation, which is druggable since 2021 with sotorasib [119]. Therefore, alternative strategies may help targeting these proteins. Intracellular antibodies are reagents that could be applied to this objective. Intracellular antibodies are protein binders that are expressed within the cells where they will interact with their target to either track [120,121], inhibit [122,123] or degrade it [124,125].

### 4.1. KRAS

**Figure 4:**
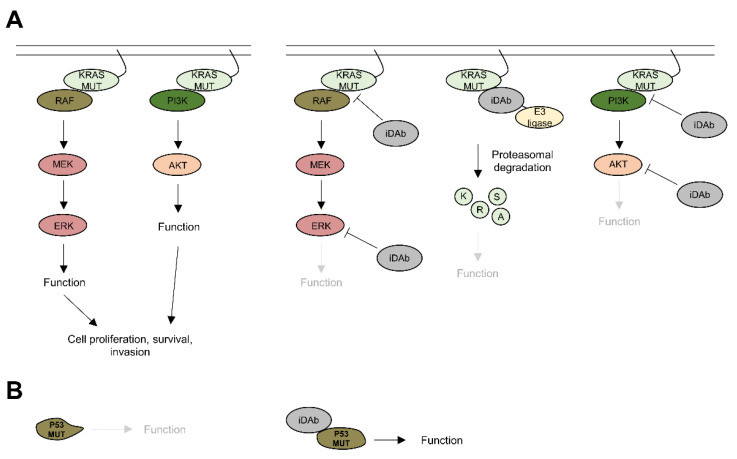
Potential of intracellular antibodies in pancreatic cancer. (**A**) Intracellular antibodies can be used to block protein–protein interactions between KRAS and its effectors, RAF or PI3K, to directly degrade KRAS by targeted protein degradation or to block downstream signalling kinases, such as ERK and AKT, to inhibit cell proliferation, survival or invasion. (**B**) Intracellular antibodies could be employed to restore the function of tumour suppressors, such as P53, by modifying their conformation.

KRAS switches between an inactive GDP-bound state and an active GTP-bound state. When mutated, KRAS persistently activates downstream signalling pathways by interacting with various effector proteins, leading to cell proliferation, survival and/or invasion [126] (Figure 4A). Hence, the first strategy was implemented by the Rabbitts group with the characterisation of an intracellular scFv that binds an RAS-GTP conformation and inhibits RAS transformation in vitro [127]. An intracellular single domain antibody (iDAb) was then developed and showed for the first time the feasibility to impede RAS/effector protein–protein interactions (PPIs) as an effective strategy to inhibit tumour growth and metastasis in vivo [123,128] (Figure 4A). However, the withdrawal of the iDAb led to a restart of the tumour growth [128], suggesting that combination therapies might be needed to effectively induce cancer cell death and avoid the apparition of resistance mechanisms. These data were further supported by the studies of the Kim lab. They developed anti-RAS-GTP intracellular full-length antibodies blocking RAS PPIs, demonstrated that resistance appeared after treating tumours with these molecules and that combination therapies were needed to overcome these resistance mechanisms [129,130].

One issue with PPI inhibitors is their mode of action that is occupancy-driven, where one inhibitor inhibits one target. Targeting protein degradation offers the advantage of working by an event-driven mode of action, which means that one degrader can deplete several targets (i.e., catalytic mechanism). Hence, the antibody-based degrader technology is a promising therapeutic strategy. This was highlighted by the functionalisation of both the anti-RAS iDAb with the UBOX domain from the CHIP E3 ubiquitin ligase and the anti-KRAS antibody mimetic binder [122] with the von Hippel–Lindau (VHL) as antibody-based degraders [125]. These RAS degraders have shown efficacy in all cell lines tested, including pancreatic cancer cells. They efficiently depleted RAS/KRAS proteins within a few hours, consequently inhibited RAS downstream signalling pathways and induced cancer cell death by apoptosis. In vivo, these degraders led to the rapid regression of mutant (K)RAS tumours, suggesting that the targeted degradation of (K)RAS is an attractive therapeutic strategy [125] (Figure 4A).

The persistent activation of mutated KRAS in PDAC leads to the activation of downstream signalling pathways, such as mitogen-activated protein kinase (MAPK) and the PI3K pathways. Therefore, targeting downstream mediators of RAS signalling combined (or not) with KRAS inhibition, for instance, could be another possibility. Several intracellular antibodies or antibody mimetics have been developed towards these RAS downstream mediators.

### 4.2. AKT

AKT is a kinase family that includes three isoforms (AKT1, 2 and 3). While AKT1 and AKT2 are ubiquitously expressed, AKT3 is predominantly found in the heart, brain and kidney [131]. AKT activation is one of the most common molecular alterations in human cancers and regulates cell proliferation and survival but also response to nutrient availability and protein synthesis, which are hallmarks of cancers [132]. Hence, several groups developed anti-AKT intracellular antibodies (Figure 4A). Pan-AKT inhibition with a scFv was previously reported, and its expression within cells led to apoptosis in vitro and in vivo [133]. Because pan-AKT inhibition can induce unwanted toxicities, a more specific inhibition of AKT isoforms was achieved, with nanobodies specifically targeting either AKT1 [134] or AKT2 [135], and it showed inhibitory effects in vitro, but additional works would be required to check their efficacy in vivo.

### 4.3. ERK

ERK1/2 is a kinase belonging to the MAPK pathway that is involved in the signal transduction into the nucleus to activate numerous transcription factors, such as FOS, JUN or MYC, that ultimately control cell proliferation [136]. Antibody mimetic binders targeting either ERK1/2 or the phosphorylated ERK1/2 were developed (Figure 4A), but their potency to inhibit cell proliferation in vitro and tumour growth in vivo needs to be assessed [137].

### 4.4. Alternative Strategies

Mutated tumour suppressors, such as TP53, can have their conformation modified and, consequently, be inactivated or have a decreased expression [138]. Therefore, restoring the activity of a mutant tumour suppressor with intracellular antibodies is an attractive therapeutic possibility that would be worth exploring. Actually, this has been achieved with scFvs targeting mutant P53 and restoring its activity in vitro [139,140,141], but this strategy was not tested in vivo. Nevertheless, small peptides targeting mutant P53 restored the WT conformation to mutant P53 with in vivo activity [142], showing the feasibility of such an approach in a preclinical setting (Figure 4B).

## 5. Conclusions and Future Directions

Pancreatic cancer is an aggressive cancer with limited treatment options that has only modest clinical responses. Therapeutic monoclonal antibodies have been successful in many cancers, but, as we discussed in this review, they also have limited efficacy in PDAC as monotherapy most likely due to the heterogeneity found in PDAC tumours. Therefore, (i) there is a need to discover a novel target for therapy, and the development of strategies, such as the surfaceome, could be advantageous. (ii) The combination of several therapeutic strategies (including mAbs) is most likely the future of PDAC treatment, with, notably, personalised medicine, such as vaccines, to overcome the heterogeneity issue [143]. Several clinical trials are ongoing with a combination of different mAbs, such as the phase I/II trial that is currently recruiting patients and employs the anti-MSLN ADC (anetumab ravtansine) with an anti-PD1 (nivolumab) and/or anti-CTLA-4 (ipilimumab) and/or gemcitabine (NCT03816358). (iii) As discussed in this review, while non-modified mAbs showed limited efficacy, their functionalisation into ADC or radiolabelled mAbs could enhance their efficacy. However, not all mAbs can be modified, particularly because their target needs to be internalised for ADC or targeted radionuclide therapy to work and not all cell surface proteins internalise. One cause of the little effect of the mAbs is the dense TME found in PDAC, which limits the tumour accessibility. Consequently, therapeutic strategies should include molecules that interfere with this TME, such as targeting a subset of cancer-associated fibroblasts (CAF) [144] or their secreted products (e.g., TGF-β) [145].

Nevertheless, using mAbs for the diagnostic or visualisation of the tumours is promising, as revealed by the development of SGM-101-modified mAb for image-guided surgery. Again, the discovery of novel biomarkers will help to increase the accuracy of tumours imaging. For instance, it would be of interest to discover specific cell surface proteins on pancreatic cancer tissue at an early stage (e.g., PanIN lesions) to improve the diagnostic/tumour imaging, but this requires the availability of patient tissue at an early stage.

One major issue with PDAC tumours is their “cold” immunogenic property, which impedes the direct use of CPIs that demonstrated great results for other cancers. While personalised vaccines might overcome this problem, other solutions are studied to make the tumours immunogenic, such as oncolytic viruses [146].

Finally, the implementation of intracellular antibodies is far from reaching the clinic yet, but this application is promising as it could directly interfere with the main oncoproteins or tumour suppressors that are deregulated in PDAC. The major hurdle to pass is the delivery inside the cells of such reagents. This is under development, with different strategies being investigated, such as viral or non-viral delivery strategies [9]. The latter is notably promising because it has been successfully employed to deliver mRNA, encoding the trimerized receptor-binding domain of the spike glycoprotein of SARS-CoV-2 with lipid nanoparticles for COVID-19 mRNA vaccine [147]. Another way of using intracellular antibodies has been developed by the Rabbitts group with the antibody-derived compound (Abd) strategy. Abd uses intracellular antibodies as a guide to select small molecules that would display the same inhibitory mechanism as the intracellular antibody. This strategy has been successfully applied to LMO2 and RAS oncoproteins [148,149,150,151,152,153], with notable antiproliferative and cytotoxic effects for the anti-RAS compounds in RAS-mutated cancer cell lines.

## Data Availability

Not applicable.
